# Prioritizing high-contact occupations raises effectiveness of vaccination campaigns

**DOI:** 10.1038/s41598-021-04428-9

**Published:** 2022-01-14

**Authors:** Hendrik Nunner, Arnout van de Rijt, Vincent Buskens

**Affiliations:** 1 Department of Sociology/ICS, Utrecht University, Utrecht, The Netherlands; 2 Centre for Complex System Studies (CCSS), Utrecht University, Utrecht, The Netherlands; 3 Department of Political and Social Sciences, European University Institute, Florence, Italy

**Keywords:** Infectious diseases, Epidemiology, Risk factors, Mathematics and computing

## Abstract

A twenty-year-old idea from network science is that vaccination campaigns would be more effective if high-contact individuals were preferentially targeted. Implementation is impeded by the ethical and practical problem of differentiating vaccine access based on a personal characteristic that is hard-to-measure and private. Here, we propose the use of occupational category as a proxy for connectedness in a contact network. Using survey data on occupation-specific contact frequencies, we calibrate a model of disease propagation in populations undergoing varying vaccination campaigns. We find that vaccination campaigns that prioritize high-contact occupational groups achieve similar infection levels with half the number of vaccines, while also reducing and delaying peaks. The paper thus identifies a concrete, operational strategy for dramatically improving vaccination efficiency in ongoing pandemics.

## Introduction

Today, two years after its global outbreak, sustained propagation of COVID-19 continues to kill thousands of people a day, inflict economic damage and allows mutations to emerge that may require the development of new vaccines. The challenge this poses is how to effectively control the virus with limited means. In this article, we draw on social network analysis to propose an easily implementable strategy for more effectively vaccinating a population.

Two decades ago, network scientists showed that in theory, the targeting of highly connected individuals should be an effective vaccination strategy when propagation networks exhibit high variability in connectivity across nodes^1,2^. Recent studies that calibrate models using data on close-range contact frequencies have demonstrated that also in the case of COVID-19, the prioritizing of individuals with many close-range contacts would dramatically increase the effectiveness of vaccination campaigns^3,4^. This is because short-range physical contact is highly unequally distributed across individuals. “Hubs” have many more contacts than other individuals, and according to contact diaries, these contacts are not shorter-lived^3^. As a result, hubs are not just more likely to get infected, but once infected, they also pass it on to more others.

Despite its promise, this strategy has remained mostly a theoretical idea^5^. A key obstacle to implementation is the ethical and practical problem of differentiating vaccine access based on a personal characteristic that is hard-to-measure and private. How does one identify high-contact individuals so that they can be targeted in vaccination campaigns? We propose the use of occupational groups as a proxy for the number of close-range contacts in a contact network. Differentiating COVID-19 policy interventions on the basis of individuals’ occupations is executable. Indeed, it has already been part of public policy in many countries, both in social distancing legislation and vaccine access, except that prioritization was not based on network analysis.

For this approach to be effective, there must be significant variability in close-range exposure between individuals working in different occupations. Clearly, occupational group is imperfect as a proxy, as people with the same job can still vary greatly in the number of short-range contacts they have. In this paper, we draw on data from a recent survey conducted at the beginning of the COVID-19 pandemic in early 2020^6^ that combines detailed occupational codes with measures of close-range contact. The survey covers six countries—China, South Korea, Japan, Italy, UK, and US—and is nationally representative of each by age, gender, and income. Information is available on contact at *under* 1 *meter distance* prior to the COVID-19 pandemic, as well as on such short-range contact during the first lockdown in Spring 2020. The data reveal substantial occupational differences, with teachers and cashiers being among the most connected and computer programmers among the least connected. To investigate whether this variability can produce significant gains when exploited in targeted vaccination programs, we used the data in two ways. First, we generated networks that have degree distributions calibrated with occupational contact data. Second, we simulated epidemics and compared the effectiveness of vaccination campaigns targeting individuals randomly or targeting occupational groups with the highest average number of social contacts.

### Related work

Since the outbreak of COVID-19 in early 2020, a plethora of scientific studies has been published from a wide variety of disciplines, such as medical sciences^7–9^, artificial intelligence and machine learning^10,11^, social sciences^12–15^, psychology^16,17^, economy^18–20^, and food and agriculture^21,22^. Our model builds in particular on a long strand of so-called compartmental models. The origins of compartmental models in epidemiology^23^, for example, go back more than a century. These models divide a population into different compartments representing disease states (e.g., susceptible, infected, recovered) and define how to progress from one compartment to another. They are a powerful tool for predicting the possible course of epidemics and the effectiveness of countermeasures^24–28^. Compartmental models have also been used for the simulation of diffusion processes on social networks, such as disease spread^29,30^, information spread^31^, and their interplay^32^. Our model likewise explicitly simulates the diffusion of an infectious disease in a social network.

Recent studies have proposed numerous network interventions^33^ for reducing the propagation of COVID-19. Some interventions seek to strategically restrict close-range contact to occur only within predetermined interaction structures so that the speed and reach of COVID-19 spread can theoretically be greatly reduced^34,35^. However, even severe social distancing policies such as full-scale lock-downs can only temporarily reduce infections and hospitalizations^36–42^, leaving large-scale vaccination as the primary vehicle for sustainable control over the SARS-CoV-2 virus. Highly effective vaccines are being mass-distributed and evidence is mounting that vaccinations do not just prevent severe cases but also greatly reduce infection^43–51^. Nonetheless, global vaccine roll-out has logistical and financial limits. We study a different kind of network intervention that seeks to minimize resources needed to achieve a certain level of epidemic control by strategically making use of network properties. Specifically, we research the prioritizing of occupational groups with workers exposed to close-range contact with large numbers of individuals.

For many diseases spreading through close-range contacts, evidence has accumulated that a small fraction of source individuals is responsible for most infections^52–58^. It is estimated that for COVID-19, between 10 and 20% of infected individuals produce 80% to 90% of new cases^59–64^. This suggests that if one could somehow identify and protect the minority of spreaders, the virus may be controlled through focused interventions at lower overall cost. While the mechanisms that underlie interpersonal variability in infectiousness are poorly understood^65–67^, it is self-evident that the more others one exposes to a given intensity and duration of short-range contact, the larger the number of new cases that one generates. One may suspect a trade-off between the number of close-range contacts and the length of such contact. Then, if the infection probability were increasing in contact length, hubs would not play a relatively less critical role. However, data from contact diaries suggest that those who meet only a handful of people on one day do not expose these others for a longer period of time than those who meet dozens of distinct people on one day^3^, reinforcing the strategic value of targeting hubs in contact networks for vaccination and other forms of infection prevention.

## Simulation model

Networks of 10,000 nodes were generated using a network formation model^30^ that allows control of degree (for details, see Methods). A genetic algorithm was used to fit the average degrees per major occupational group (according to the SOC codes from the US bureau of labor statistics) reported for times prior to the epidemic. Table 1 shows the numbers for reported mean degrees by major occupational group^6^. Recent US labor market numbers were taken to set occupational group size^68^. *Office and Administrative Support Occupations*, for example, were the largest group containing 12.74% of the entire labor market, and thus our generated networks included the same percentage of nodes for this occupational group. A cross-sectional survey study on social contacts in GB among 5000 respondents^69^ estimated the average proportion of closed triads in contact networks to be about 0.46, while reducing with age. Accordingly, we varied clustering in a range around that value, at 0.3, 0.4, and 0.5. Occupational group homophily was varied to cover scenarios with no (0.0), medium (0.4), and high (0.8) probabilities of ties between nodes from the same occupational group.

**Table 1:** Mean degrees per major occupational group for empirical networks at time points prior to (normal) and during the first COVID-19 lockdown in Spring 2020.

	Normal	Lockdown
Healthcare Practitioners and Technical Occupations	20.17	5.19
Personal Care and Service Occupations	12.82	4.58
Educational Instruction and Library Occupations	12.76	2.61
Legal Occupations	8.28	0.92
Management Occupations	5.84	1.32
Sales and Related Occupations	5.59	3.3
Healthcare Support Occupations	5.44	3.7
Food Preparation and Serving Related Occupations	5.32	1.57
Transportation and Material Moving Occupations	5.31	2.69
Life, Physical, and Social Science Occupations	4.63	3.55
Office and Administrative Support Occupations	4.42	1.94
Building and Grounds Cleaning and Maintenance Occupations	3.93	1.26
Installation, Maintenance, and Repair Occupations	3.76	2.83
Business and Financial Operations Occupations	3.66	1.52
Construction and Extraction Occupations	3.6	1.99
Architecture and Engineering Occupations	3.47	1.88
Arts, Design, Entertainment, Sports, and Media Occupations	3.23	2.51
Production Occupations	2.87	2.91
Computer and Mathematical Occupations	2.85	1.21
Community and Social Service Occupations	2.84	1.08
Unemployed	2.34	0.96
Farming, Fishing, and Forestry Occupations	2.15	1.76
Retired	2.13	0.87
Protective Service Occupations	1.12	1.05

For each combination of clustering (3 values) and homophily (3 values), we selected the 10 best fitting networks. Based on these 90 normal networks, another 90 lockdown networks were generated by severing ties between nodes. Ties were severed based on the average contact number reduction reported for the two connecting nodes’ occupational groups (Table 1). This procedure lasted until the empirical average degrees of the occupational groups were achieved. Consequently, we ended up with a total number of 180 networks. Detailed descriptive statistics on network composition and fitting of average degrees to occupational groups can be found in respectively Tables S1 and S2–S5 in the supplementary information.

**Figure 1:**
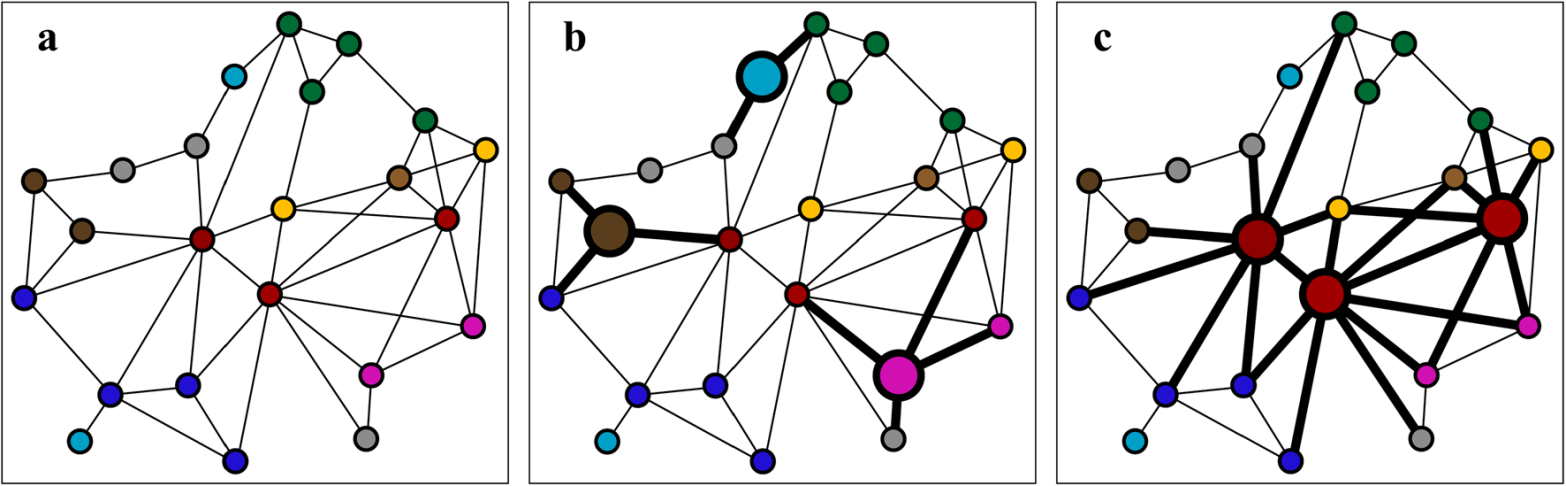
Vaccination campaign scenarios. (**a**) Baseline scenario without vaccinations, (**b**) random distribution of vaccines, and (**c**) targeted distribution of vaccines based on occupational group membership in descending order of average number of social contacts. Nodes represent individuals and colors represent occupational group membership (e.g., red: *Healthcare Practitioners and Technical Occupations* with high average degree, brown nodes: *Office and Administrative Support Occupations* with low average degree). Enlarged nodes represent immunized individuals. Thick ties represent social connections of immunized nodes, and are therefore ruled out as possible transmission routes.

To assess the effectiveness of different vaccination campaigns, we simulated epidemics under three different conditions shown in Fig. 1. In the *baseline* condition (a), no vaccinations were given, and thus all nodes remained susceptible. The baseline condition therefore provides a benchmark for judging the effect of vaccination campaigns on epidemics. The two vaccination campaigns differ in the way nodes are selected for the administration of vaccinations. In the *random* condition (b), randomly selected nodes were vaccinated irrespective of occupational group membership. In the *targeted* condition (c), nodes were vaccinated based on occupational group membership and in descending order of the reported average number of social contacts (i.e., 1. Health Practitioners and Technical Occupations, 2. Personal Care and Service Occupations, 3. Educational Instruction and Library Occupations, etc. in the normal networks; and 1. Health Practitioners and Technical Occupations, 2. Personal Care and Service Occupations, 3. Healthcare Support, etc. in the lockdown networks; see Table 1).

**Figure 2:**
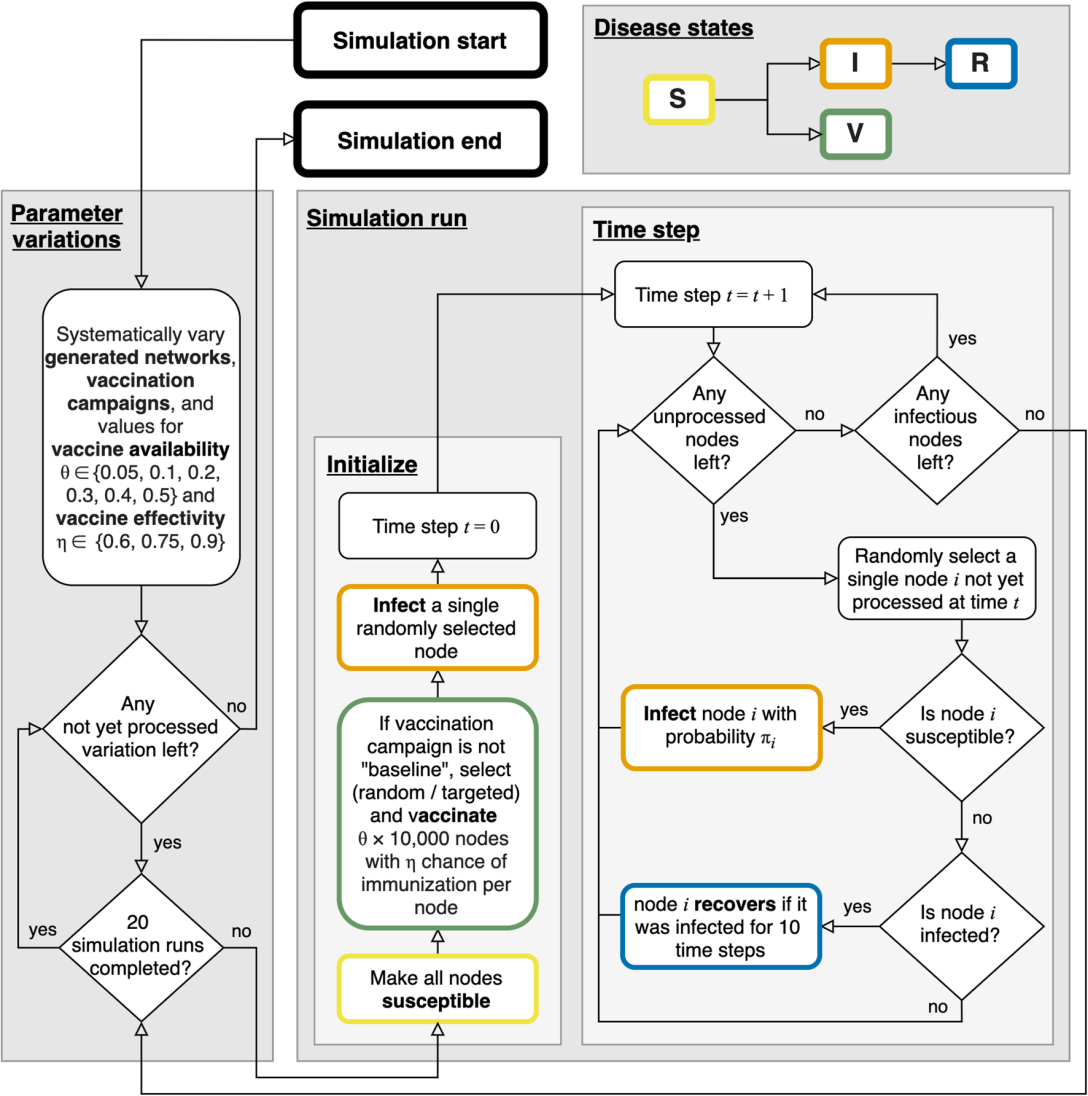
Flowchart of the simulation.

A flowchart for how we simulated disease transmission and intervention is given in Fig. 2. In addition to varying the input networks (normal vs. lockdown, clustering, occupational group homophily), we varied the availability of vaccines for the population (5%, 10%, 20%, 30%, 40%, 50%) and vaccine effectiveness in terms of probability of immunization (0.60, 0.75, 0.90). Note that the latter entails both the probability to prevent sickness and the probability that the disease is spread further. 20 simulation runs were performed for each of the 180 networks, 3 vaccination campaign conditions, and 6 vaccine availability percentages as well as 3 vaccine effectiveness controls (non-baseline conditions only), resulting in a total number of 133200 simulated epidemics.

Each simulation run was initiated by distributing vaccinations to an entirely susceptible population. Whether a node was immunized depended on whether the node was selected for vaccination (vaccination campaign condition and vaccine availability) and whether the vaccination was successful (vaccine effectiveness). In contrast to immunized nodes, unsuccessfully vaccinated nodes remained susceptible. Note that we neglect the possible spread of the disease through vaccinated people who do not develop symptoms.

In a second step, a randomly selected node was infected (*index case*). The remainder of a simulation run consisted of discrete time steps to compute disease transmission between infectious and susceptible nodes and recovery events of infected nodes. That is, whether a node *i* got infected depended on the probability of disease transmission per single contact ($\gamma = 0.15$) and the number of infectious contacts of node *i* ($n_{i_{I}}$):

1 $$\begin{aligned} \pi _{i} = 1 - (1 - \gamma )^{n_{i_{I}}} \end{aligned}$$

Infectious nodes recovered after 10 time steps and could not get infected a second time. A simulation run ended when no infectious nodes were left. Detailed descriptive statistics on network compositions, index cases, epidemics, and counter measures can be found in Table S1 in the supplementary information.

## Results

### Comparison of vaccination campaigns

**Figure 3:**
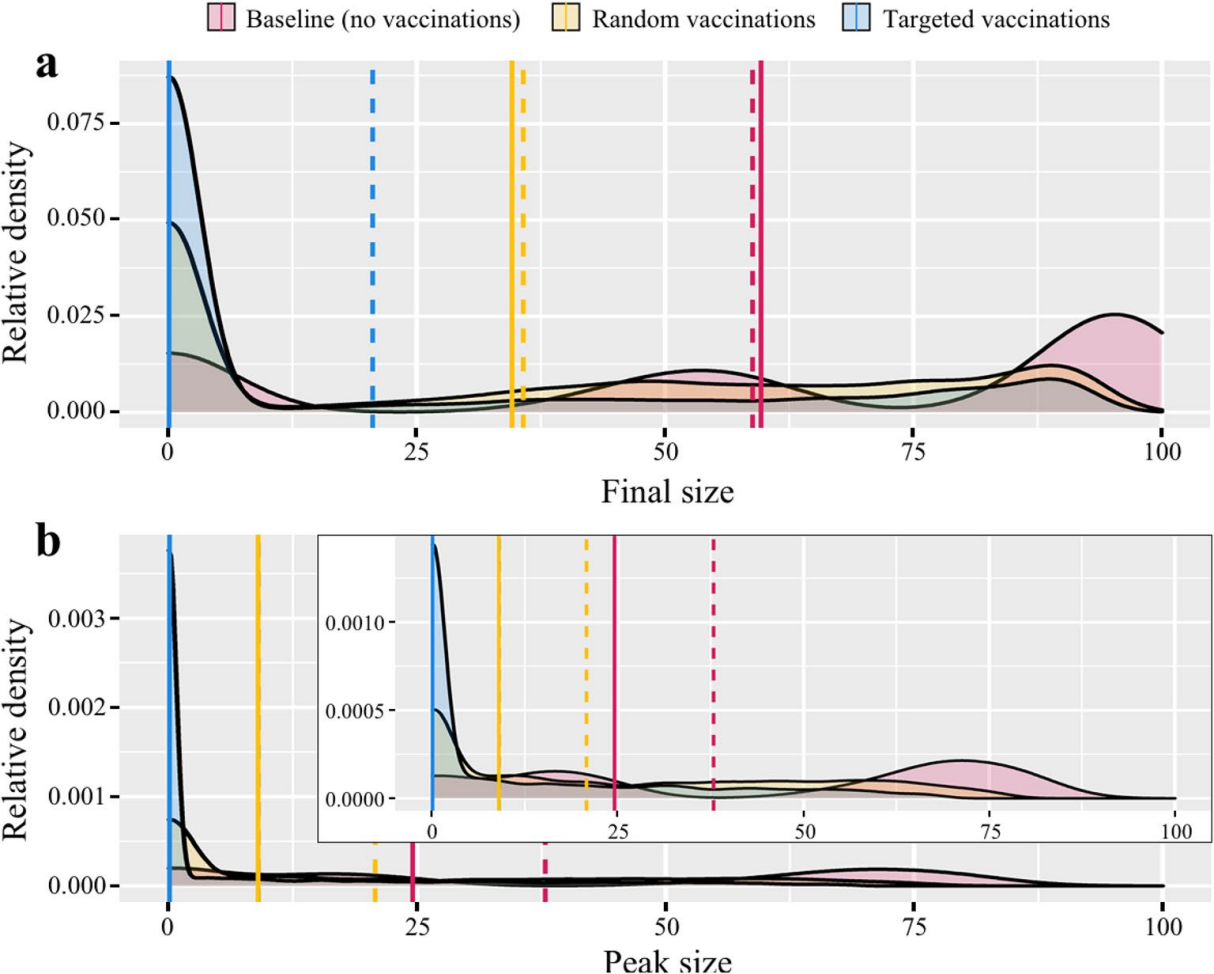
Densities of final and peak size of epidemics. Relative densities of final size (**a**) and peak size (**b**) of epidemics by vaccination campaign. Both measures are reported as percentage of the population (final size: percentage of cumulative infected nodes; peak size: maximum percentage of simultaneously infected nodes). Dashed lines show mean, solid lines show median values. Note that mean peak size in the Targeted condition is largely covered by median peak size in the Random condition.

Figure 3 shows across all simulation runs, separately for each vaccination scenario, the distributions of two commonly studied measures of epidemic control: (a) final size and (b) peak size. Final size is the percentage of nodes that have been infected over the entirety of a simulated epidemic. Peak size describes the maximum percentage of simultaneously infected nodes per epidemic. To increase the resolution of differences between the conditions, the inset of plot (b) shows peak sizes in epidemics involving a minimum of at least two simultaneously infected nodes. Each plot shows the relative frequencies of one measure per vaccination campaign (baseline—red, random—yellow, targeted—blue). Dashed lines depict mean values, and solid lines median values per vaccination campaign.

Our simulations suggest that independently of network composition, vaccine availability, and vaccine effectiveness, targeting high contact occupations is much more effective than random distribution of vaccines. While the random campaign produces average final sizes of about 35% infected nodes (both mean and median), the targeted campaign produces mean final sizes of only 20% infected nodes and even prevents most epidemics entirely (median final size close to 0%). A similar picture is drawn regarding peak size. While random distribution of vaccines produces epidemics with about 10% (median) to 20% (mean) simultaneously infected nodes, the majority of epidemics in the targeted distribution do not show notable peaks at all (median close to 0%).

**Table 2:**
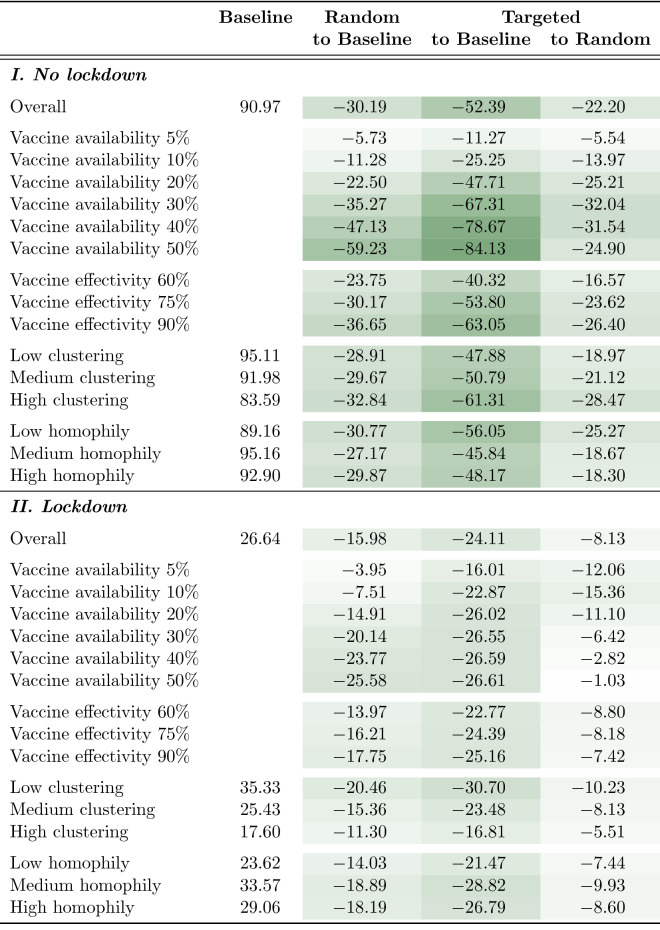
Mean final size of baseline condition (2nd column) and difference by test condition in percent points.

*Notes:* Raw numbers are provided in Table S6 in the supplementary information.

Table 2 shows that, independent of input parameter variations, targeted distribution of vaccines is substantially more effective in reducing infection numbers than random distribution. Specifically, targeted distribution of vaccines achieve about the same reduction of infection numbers with only *half* the number of vaccines. That is, targeted vaccination of 5% of the population reduces final size by 11%, while the same reduction in the random condition requires 10% vaccine coverage. The same applies to 10% and 20% vaccine availability in the targeted condition, which require 20% and 40% vaccine coverage in the random condition, respectively.

The effect of vaccine availability is even more striking for the lockdown networks (lower half of Table 2). That is, targeted vaccination of 20% of the population can bring the epidemic to a halt (reduction of 26.02% points from 26.64 in the baseline condition). To achieve the same with random distribution of vaccines, two-and-a-half times the number of vaccines is required, as about half of the population would need to be vaccinated. In sum, with or without lockdown, targeted vaccination greatly improves campaign effectiveness.

A similar picture is drawn for vaccine effectivity. For both network types (no lockdown, lockdown), and all other parameters considered equal, targeted distribution of vaccines reduces the number of infections significantly stronger than random distribution of vaccines.

These main results are broadly robust across network parameters. Table 2 shows effects of network composition on the final size of epidemics. Clustering has been divided into three categories: networks at the lower end (0.4), the upper end (0.6), and in the middle (0.5) of the clustering range reported for contact networks^69^. The simulations show that while targeted intervention is more effective at all clustering levels, the more clustered the network, the greater the relative gains vis-à-vis random. Occupational group homophily is also divided into three categories. The *low homophily* category contains the networks without consideration of occupational group for tie creation. The *medium homophily* category contains networks that used a probability of 0.4, while the *high homophily* category contains networks that used a probability of 0.8 for the creation of ties between nodes from the same occupational group. Because of the redundancy of occupation-targeted intervention under high occupational homophily, we find that when homophily is increased the effectiveness of targeted versus random intervention is somewhat reduced in the non-lockdown networks.

### Dynamics of epidemics

**Figure 4:**
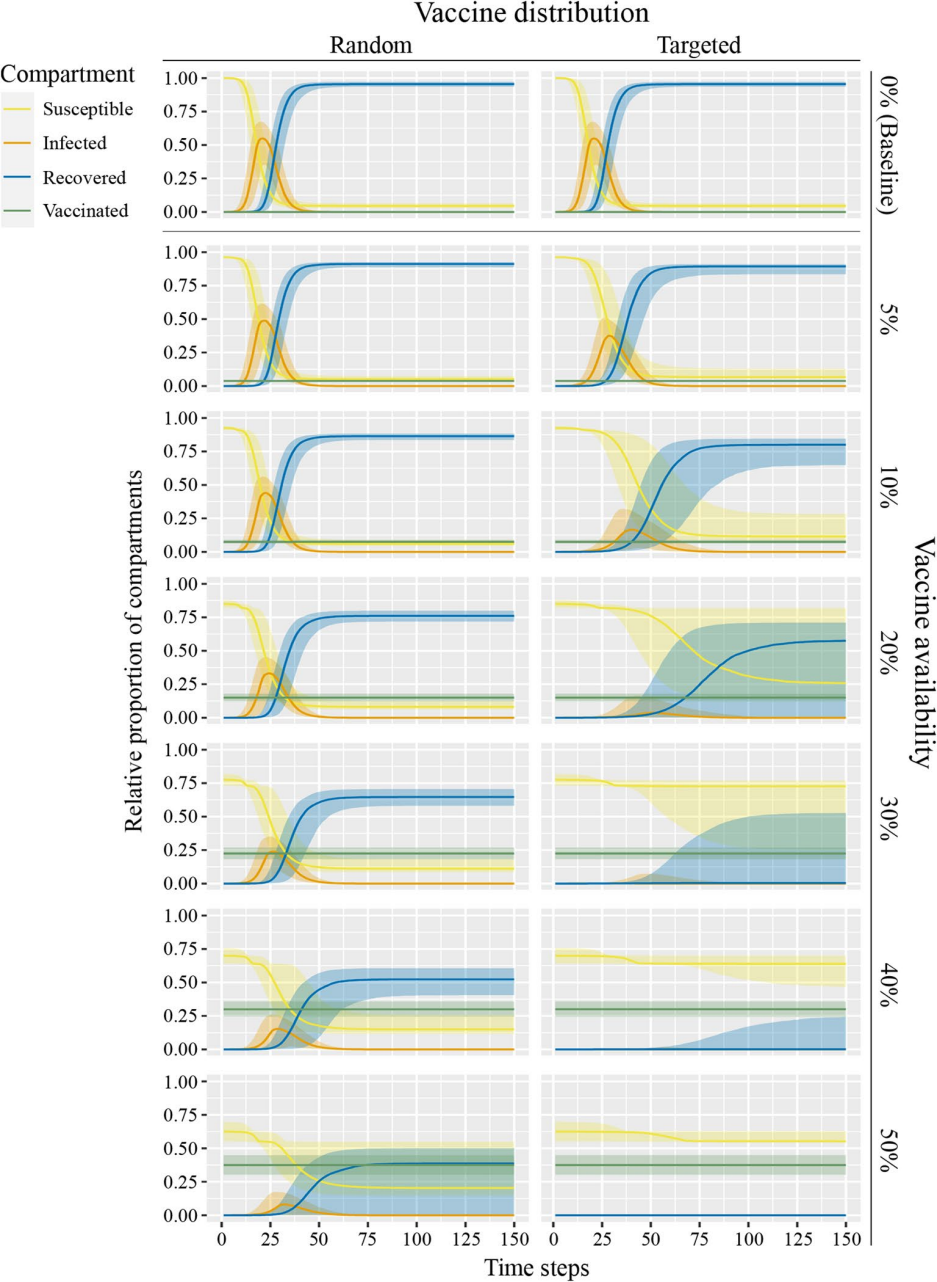
SIRV plots for vaccine availability per vaccination campaign condition. Solid lines show median proportion (y-axis) of susceptible (yellow), infected (orange), recovered (blue), and successfully vaccinated/immunized (green) agents over the first 150 simulated time steps. Ribbons show interquartile range.

Figure 4 visualizes the effects of vaccine availability and type of distribution on the temporal progression of epidemics. Depicted is a comparison of the average course of epidemics between the three vaccination campaign conditions (first row: Baseline; first column: Random; second column: Targeted) and the availability of vaccines (rows). Each plot shows colored lines for the median proportion of susceptible (yellow), infected (orange), recovered (blue), and vaccinated (green) nodes over time. Ribbons show the variability (interquartile ranges) of these results.

As before, we find that with only half of the vaccines the same reduction of final size can be achieved through prioritization of high-contact occupations: When comparing the plots for 20% vaccine availability in the targeted distribution condition (column 2, row 4) and 40% vaccine availability in the random distribution condition (column 1, row 6) we observe a similar end point for the number of recovered nodes (i.e., final size). Additionally, we can see that the shape of the curve of infected nodes differs. That is, the peak in the targeted distribution condition is at an average of 15.22%, while the peak in the random distribution condition is at an average of 23.60% (see Table S7 in the supplementary information). Furthermore, the epidemic in the random distribution condition requires on average 71.81 time steps from the first infection until the last infected node recovers. In the targeted distribution condition, a similar number of infection and recovery events occur in 101.85 time steps (see Table S9 in the supplementary information), thus creating a flatter curve of infected nodes.

In summary, our simulations suggest that distribution of vaccines prioritizing societal groups with high contact rates (here: occupations) effectively and consistently reduces final size, peak size, and slow down the spread of a disease, largely independently of input parameter variations. That is, while similar numbers of infections can be achieved with only half the number of vaccines, targeted vaccinations can also *flatten the curve* more effectively than random distribution of vaccines.

## Discussion

These results bring the theoretical strategy of targeting people with many close-range contacts in vaccination campaigns one step closer to real-world implementation. We have long known from theoretical diffusion studies that targeting interventions at hubs in social networks should reduce the spread of infectious diseases that are passed on through person-to-person contact^1,2^. Furthermore, recent model studies suggest that prioritizing individuals with many close-range contacts in the case of COVID-19 would dramatically increase the effectiveness of vaccination campaigns^3,4^. Although all these studies, including our own, promise large efficiency gains through network targeting, they are hardly used for disease control. This is because implementation of network targeting has been impeded by the issue of how to identify people with many contacts in a practical manner. Deriving contact data from digital trace data poses privacy concerns. Random neighbor sampling^70,71^, whereby randomly nominated friends are vaccinated, may also work because of the statistical tendency for one’s friends to have more friends than oneself has^72^. However, the lack of familiarity with selecting recipients of a life-saving public health intervention through such unorthodox methods may raise practical, legal, and ethical challenges.

By contrast, policies that intervene based on target individuals’ occupations are already used widely. During the COVID-19 pandemic in various countries, certain occupations saw mandatory closures, while other sectors of the economy were allowed to remain open. In the vaccination campaigns in most Western countries, certain occupations, such as doctors and teachers, were given preferential access to vaccines. The Centers for Disease Control and Prevention (CDC) already recommends the prioritization of *essential workers* who “ensure the continuity of critical functions in the United States” with those higher in order who have higher risks of exposure to SARS-CoV-2^73^. Similar prioritization can be found in other countries’ vaccination strategies around the world. Many strategies, however, do not consider prioritization of occupational groups beyond essential occupations or healthcare occupations.

In this paper, we claim that expanding vaccination strategies to focus more on the number of contacts per occupational group, can reduce the final size of an epidemic by nearly twice as much (52% decrease) compared to strategies neglecting contact rates (30% decrease). In some scenarios, targeted vaccination requires only half the number of vaccines of non-targeted vaccination to achieve similar final sizes with later and lower epidemic peaks. Furthermore, the paper has shown that the positive effect of targeted vaccination is independent of vaccine effectivity and can be increased by stronger network clustering and lower occupational group homophily. Thus, maintaining social distancing policies to limit social contacts to close family and friends (increasing clustering) and working remotely (decreasing occupational group homophily) during targeted vaccination roll-out may help to bring an epidemic to a quicker end.

Currently, the choice for prioritizing people with certain occupations is often made based on how important these occupations are for society and the vulnerabilities individuals in these occupations are exposed to. Furthermore, age and preexisting conditions that increase the vulnerability of specific people play an important role in the planning of COVID-19 vaccination strategies. We do not claim that the maintenance of essential societal functions and the protection of the most vulnerable should not be given the highest priority. We do claim, however, that it is important to also consider the number of relations people in an occupation have and thus their role in spreading an epidemic further. That is, people with many relations are not only more likely to get infected themselves, but are responsible for causing larger numbers of secondary cases. Immunizing persons in high contact occupations therefore has the potential to increase effectiveness of vaccination campaigns, as more people can be (indirectly) saved from infections. This becomes all the more important when vaccine availability is limited. Moving forward, an important question is how to integrate contact numbers into existing vaccination strategies. Based on our findings, we propose that occupational groups can function as a reasonably effective proxy for such an extension.

Our study has several important limitations. First, while we were able to reproduce the mean measured contacts per occupation based on the available data on these occupations, these are only estimates and for some occupations based on relatively few observations. Moreover, small sample sizes for single occupations and minor occupational groups forced us to use major occupational groups. While this approach allows an easy-to-implement strategy for policy design, it neglects, however, that the number of relations may vary between occupations within the same group. In particular, we see that the variance in numbers of relations within occupations is larger in the empirical data than in the simulated networks. The reason is that we focused on matching average numbers of relations with our network generation procedure. Furthermore, matching variance is not straightforward. In addition, given the numbers of observations and the numbers of the relations reported in the data, it can be inferred that the variances are not well estimable based on the empirical data and outliers might cause variances to seem larger than they actually are.

The data we used also showed that differences in contact numbers between countries exist. Average contact numbers in the UK before the pandemic (9.48 contacts per day), for example, were almost 3.5 times as large as for Korea (2.77 contacts per day). Our results therefore show an average effect that may differ between countries. Furthermore, we do not consider age structures or household compositions. Thus, results are focused on occupational networks only, neglecting the role children may play in the continuation of an epidemic. Lastly, we do not take advantages or disadvantages of strategies into account that go beyond the studied network effects. Prioritizing specific job categories, for example, may have additional effects on the course of an epidemic (e.g., medical sector, tourism industry).

While we are confident that the main result of a large efficiency gain from targeted vaccination is robust, this gain may differ in magnitude when applied to specific diseases. To adapt the model to COVID-19, for example, some details, such as probability of transmission per contact, recovery times, and finer resolution of compartments, need to be modelled more precisely. Furthermore, we assume that successfully vaccinated people cannot get, nor spread the disease anymore. Although it is still under discussion to what extent vaccinated people remain potential transmitters of COVID-19 and thus reduce the effect of targeted vaccine distribution, it is now clear that vaccinations do not just prevent severe cases but greatly reduce infection^52–58^. These effects, however, will depend on viruses and variants that emerge and the vaccines that are developed to combat them.

In conclusion, when severe social distancing measures do not suffice, vaccination remains the most effective weapon against an epidemic outbreak. Although we have long known that immunizing high contact individuals can reduce the spread of infectious diseases, these measures have hardly been used for disease control. Our study suggests that using high-contact occupations as a readily available proxy for targeted vaccination campaigns can significantly increase the effectiveness of vaccine roll-out, while avoiding some pitfalls impeding implementation (e.g., privacy concerns, practical, legal, and ethical challenges).

## Methods

The methods presented here provide a higher-level overview. The aim is to promote understanding of the simulation procedures and to enable putting the results into context. For a more detailed and formal description of the methods, including all equations, pseudocode of algorithms, and parameter settings, please refer to the section Supplementary Methods in the supplementary information.

### Network formation model and simulation

The networks used as input for the simulation of epidemics and the simulation of epidemics are based on a specific model case^74^ of the *Networking during infectious diseases model (NIDM)*^30^. The network formation model is based on the idea that social ties provide utility^75,76^. Furthermore, this utility can be maximized by changing the position someone takes in the network. Utility in the NIDM describes personal well-being from the perspective of each node *i* and is the difference between benefits of social ties (social capital, comfort, sense of belonging, etc.) and costs to maintain these ties (time, effort, etc.):

2 $$\begin{aligned} U_{i} = b_{1} \cdot t_{i} + b_{2} \cdot \left( 1 - 2 \cdot \frac{\left| x_{i} - \alpha \right| }{\max \left( \alpha , 1 - \alpha \right) } \right) - c_{1} \cdot t_{i} + c_{2} \cdot t_{i}^{2} \end{aligned}$$

$b_{1}$ is the immediate benefit for the number of ties $t_{i}$ and is discounted by the immediate costs $c_{1}$ and the marginal costs $c_{2}$ for the number of ties. Note that variations of $c_{2}$, while keeping parameters constant (here: $b_{1} = 1.0$ and $c_{1} = 0.2$), allow controlling the optimal number of ties per node. A setting of $c_{2} = 0.05$, for example, translates into an optimum of 8 ties per node, while $c_{2} = 0.1$ creates an optimum of 4 ties per node. The benefit of network positions is furthermore dependent on whether the actual proportion of closed triads $x_{i}$ matches the optimal proportion of closed triads $\alpha$ and how much weight $b_{2}$ there is on this part of the equation. Consider, for example, node A having three ties (AB, AC, AD), which implies three possible closed triads node A is part of (AB-AC-BC, AB-AD-BD, AC-AD-CD). Furthermore, consider $\alpha$ set to a value of 0.33. It follows that the optimum for node A would be to have only one of the three possible closed triads.

Agent-based simulations^77^ were used to generate the networks based on the data with normal (prior to lockdown) degree distribution. Starting from an empty network, agents maximize individual utility based on Eq. (2) by either creating or severing ties to other agents. Simulation parameter $\omega$ allows furthermore to control the proportion of ties between agents from the same occupational group. That is, corresponding to the concept of baseline homophily^78^, the simulation provides more opportunity to meet for agents sharing similar traits the higher the setting for $\omega$.

### Calibration of network structure with empirical data

Networks of 10,000 nodes were generated using the NIDM simulation and fitted to empirical data using a genetic algorithm and a lockdown generation algorithm. Empirical data to define target values of the generated networks consisted of three sources. First, employment numbers reported by the U.S. Bureau of Labor Statistics^68^ for major occupational groups according to the *Standard Occupational Classification (SOC)* system^79^. These data were used to assign a major occupational group to each agent, with a probability according to the group’s proportional size. Second, mean degree per major occupational group collected in a six-country survey on COVID-19 and reporting contact numbers before the pandemic and during the first lockdown in Spring 2020^6^ (see Table 1). Third, network clustering ($\alpha \in \{0.3, 0.4, 0.5$}) as collected in a cross-sectional study of social contacts in England, Scotland, and Wales^69^. Due to lack of empirical data, occupational group homophily was varied to realize scenarios without ($\omega = 0.0$), with medium ($\omega = 0.4$) and with strong ($\omega = 0.8$) assortative mixing.

A genetic algorithm was used to find initial settings for the average number of ties per occupational group ($c_{2}$) and the degree of network clustering ($\alpha$) that match the target values for mean degree and clustering best. Note that occupational group homophily was not considered, but varied explicitly due to the lack of empirical data. The algorithm consisted of six generations for each of the nine systematically varied parameters (3 for clustering, 3 for homophily). Each generation consisted of a number of model realizations with varying parameter settings for marginal costs per occupational group ($c_{2}$) and optimal proportion of closed triads ($\alpha$). The initial generation consisted of 4 simulations with parameter settings according to the empirical data. At the beginning of each of the following five generations, the four best fitting model realizations were selected as parents (the lowest percentage error to the target values). For each of the six possible pairs of parents, two offspring model realizations were created. That is, parameter settings for marginal costs per occupational group and clustering were randomly selected from one of the parents (gene selection). If the parameter settings created outcomes that deviated more than 2% from the target values, the parameter settings were varied randomly within a range between 0.0 and the percentage error for the according value (gene mutation). The new offspring was used to generate a network in a subsequent NIDM simulation. Each simulation lasted until the fitness (cumulative percentage errors between target and realized values for average degrees per occupational group and network clustering) did not improve for 5 consecutive rounds. A single combination of the explicitly varied parameters (prior to lockdown contacts, 3 settings for clustering, 3 settings for homophily) thus created $4 + 5 \cdot 6 \cdot 2 = 64$ networks, while the entire procedure resulted in $3 \cdot 3 \cdot 64=576$ networks. Finally, we selected the 10 best fitting networks for each parameter combination of clustering and homophily, resulting in 90 networks with normal (prior to lockdown) degree distributions.

Lockdown generation was realized by pruning the 90 previously generated networks. That is, network ties were severed based on the reduction of contacts during lockdown per occupational group. For every network tie, the probability of severing the tie depended on two aspects. First, both nodes have not reached the target lockdown degree of their corresponding occupational group. Second, the probability to sever a tie depended on the reduction of contacts during lockdown^6^. Consider two nodes are connected by a tie. Node 1 belongs to the group *Legal Occupations*, which showed an average reduction of contacts from 8.28 to 0.92 (88.89%). Node 2 belongs to the group *Healthcare Support Occupations*, which showed an average reduction of contacts from 5.44 to 3.70 (31.99%). Node 1 has 6 ties, while Node 2 has 4 ties. Thus, both nodes have more ties than the average node in their occupational group during lockdown, and the tie between the nodes is severed with a probability of $\frac{88.89 + 31.99}{2} = 60.44\%$. Lockdown generation stopped when, for every pair of nodes, at least one node reached the lockdown degree.

In summary, a total number of 180 networks were used as input for the simulation of epidemics. This number consists of the 10 best fitting networks for each of the 9 systematic parameter variations (3 for clustering, 3 for homophily) and average degrees reported prior to the first COVID-19 lockdown in Spring 2020; and the same number of lockdown networks generated from these networks.

### Technical setup and runtimes

Simulations were run on a MacBook Pro 13$''$, early 2013, with an Intel Core i5 Dual-Core processor running at 2.6 GHz, using 8 GB DDR3 RAM at 160 MHz. At the time of the simulations, the computer ran on macOS Catalina version 10.15.7 (19H524). The simulation was programmed in Java 8 with GraphStream v1.3^80^ used for graph handling. The Java code was executed using Eclipse v4.18 and Java compiler v1.8.0_91. For analyses, we used R v4.0.4^81^ with *ggplot2*^82^ for data visualization.

All simulations ran in 8 parallel threads. Runtimes are reported as combined totals. The network generation and fitting process for the normal (prior to lockdown) networks took 222 h and 24 min. Network pruning to generate the lockdown networks took 239 h and 41 min. Simulation of epidemics took 212 h and 27 min.

## Supplementary Information

Supplementary Information.

## Data Availability

The simulated network and epidemics data are available in the GitHub repository, https://github.com/hnunner/NIDM-simulation (version: v4.2.1., commit: 1707a0b, https://doi.org/10.5281/zenodo.5257528). The six country survey data of close-range contact including occupational codes^6^ can be publicly accessed at https://osf.io/aubkc/.

## Code availability

The Java source code to generate the data, and the R script to analyze the data are available under the GPLv3 license in the GitHub repository, https://github.com/hnunner/NIDM-simulation (version: v4.2.1., commit: 1707a0b, DOI: https://doi.org/10.5281/zenodo.5257528). Detailed instruction how to reproduce all simulations and analyses can be found in the release notes of version v4.2.1 on GitHub and Zenodo.
