# Supplementary material for: Prioritizing high-contact occupations raises effectiveness of vaccination campaigns
Source: Sci Rep. 2022 Jan 14;12:737. doi: 10.1038/s41598-021-04428-9 (PMC8760242; doi:10.1038/s41598-021-04428-9)
Supplement: Supplementary file 1 — Supplementary Information. [file 41598_2021_4428_MOESM1_ESM.pdf]

Prioritizing high-contact occupations raises effectiveness of vaccination campaigns

– Supplementary information –

Hendrik Nunner[1,2,*], Arnout van de Rijt[3], and Vincent Buskens[1,2]

[1]Department of Sociology/ICS, Utrecht University,
Utrecht, The Netherlands

[2]Centre for Complex System Studies (CCSS), Utrecht University,
Utrecht, The Netherlands

[3]Department of Political and Social Sciences, European University Institute,
Florence, Italy

[*]Corresponding author – Email: h.nunner@uu.nl

December 28, 2021

Supplementary Results

Table S1 reports detailed descriptive statistics on the properties of input networks (section I.), network position of initially infected nodes/*index cases* (section II.), course of epidemics split by normal vs. lockdown networks (section III.), and course of epidemics split by counter measures (section IV.).

Tables S2 – S5 report how well size and degree of occupational groups in the generated networks match the empirical data.

Table S6 reports the average final sizes of epidemics across conditions (columns) and control variables (rows). These data are the basis for the differences of final size between the baseline and test conditions presented in Table 2 of the main text.

Table S7 reports the average peak sizes of epidemics across conditions (columns) and control variables (rows). Table S8 reports the average peak sizes of epidemics of the baseline condition (column 2) and differences by test condition in percent points (columns 3 – 5), similar to differences of final size reported in Table 2 of the main text.

Table S9 reports the average duration of epidemics across conditions (columns) and control variables (rows). Table S10 reports the average duration of epidemics of the baseline condition (column 2) and differences by test condition in percent points (columns 3 – 5), similar to differences of final size reported in Table 2 of the main text.

Table S1: Descriptive statistics of input networks for the simulations (observations: 133 200).

	**Mean**	**SD**	**Median**	**Min**	**Max**	**Skew**
I. Network						
I.I. Normal						
Av. degree ($\mathcal{D}_G$)	4.92	0.21	4.92	4.46	5.3	−0.18
Clustering ($\mathcal{C}_G$)	0.38	0.1	0.4	0.21	0.56	−0.18
Av. path length ($\mathcal{L}_G$)	7.72	0.82	7.7	6.38	9.97	0.32
Assortativity, occupation ($\mathcal{A}^p_G$)	0.12	0.05	0.09	0.06	0.2	0.29
I.II. Lockdown						
Av. degree ($\mathcal{D}_G$)	2.1	0.02	2.1	2.06	2.16	0.52
Clustering ($\mathcal{C}_G$)	0.12	0.03	0.11	0.06	0.18	0.16
Av. path length ($\mathcal{L}_G$)	13.83	1.55	14.01	11.28	18.31	0.11
Assortativity, occupation ($\mathcal{A}^p_G$)	0.07	0.02	0.06	0.04	0.11	0.39
II. Index case						
II.I. Normal						
Degree	4.53	3.57	4	1	85	3.96
Clustering	0.39	0.26	0.33	0	1	0.3
Closeness	0.96	0.12	0.98	0	1	−7.58
Assortativity (occupation)	0.09	0.18	0	0	1	2.4
II.II. Lockdown						
Degree	1.98	1.76	2	0	26	1.73
Clustering	0.12	0.26	0	0	1	2.41
Closeness	0.44	0.32	0.64	0	0.74	−0.6
Assortativity (occupation)	0.06	0.18	0	0	1	3.69
III. Epidemic						
III.I. Normal						
Final size	50.8	35.86	62.11	0.01	98.24	−0.42
Duration	72.24	46.4	72	10	484	1.11
Epidemic peak size	2908.22	2466.26	2886	1	8395	0.22
III.II. Lockdown						
Final size	7.14	15.34	0.03	0.01	61.95	1.96
Duration	46.3	60.5	16	10	511	1.85
Epidemic peak size	193.57	460.08	3	1	2716	2.64
IV. Counter measures						
IV.I. Vaccinations						
Vaccinated (proportion)	0.25	0.16	0.2	0	0.5	0.16
Immunized (proportion)	0.19	0.13	0.18	0	0.46	0.37
IV.II. Baseline						
Final size	58.81	39.63	59.64	0.01	98.24	−0.51
Duration	66.73	41.91	63	10	208	0.49
Peak size	3787.89	3188.37	2454.5	1	8395	0.05
IV.III. Random vaccine distribution						
Final size	35.72	35.11	34.58	0.01	94.85	0.27
Duration	62.85	49.21	63	10	394	0.93
Peak size	2077.23	2425.01	899	1	7978	0.78
IV.IV. Vaccine distribution prioritizing occupational groups by largest degree						
Final size	20.56	32.52	0.06	0.01	94.35	1.18
Duration	55.27	61.39	22	10	511	1.64
Peak size	900.29	1682.96	5	1	7130	1.88

Table S2: Comparison of occupational groups in empirical versus generated networks, part 1.

	**Mean**	**SD**	**Median**	**Min**	**Max**	**Skew**
Architecture and Engineering Occupations						
Share of the labor market (empirical)	0.01					
Share of the labor market (generated)	0.01	0	0.01	0.01	0.02	0.09
Degree, normal (empirical)	3.47	8.99	0	0	150	8.35
Degree, normal (generated)	3.6	0.9	4	0	6	−1
Degree, lockdown (empirical)	1.88	6.31	0	0	100	8.73
Degree, lockdown (generated)	1.93	1.21	2	0	5	0.26
Arts, Design, Entertainment, Sports, and Media Occupations						
Share of the labor market (empirical)	0.01					
Share of the labor market (generated)	0.01	0	0.01	0.01	0.02	−0.19
Degree, normal (empirical)	3.23	11.03	0	0	200	10.5
Degree, normal (generated)	3.32	0.82	3	0	5	−0.85
Degree, lockdown (empirical)	2.51	21.18	0	0	500	20.67
Degree, lockdown (generated)	2.58	1.04	3	0	5	−0.39
Building and Grounds Cleaning and Maintenance Occupations						
Share of the labor market (empirical)	0.03					
Share of the labor market (generated)	0.03	0	0.03	0.02	0.03	0.22
Degree, normal (empirical)	3.93	15.38	0	0	100	5.46
Degree, normal (generated)	3.94	0.98	4	0	6	−1.04
Degree, lockdown (empirical)	1.26	4.95	0	0	60	9.41
Degree, lockdown (generated)	1.3	1.12	1	0	6	0.72
Business and Financial Operations Occupations						
Share of the labor market (empirical)	0.04					
Share of the labor market (generated)	0.04	0	0.04	0.04	0.05	−0.09
Degree, normal (empirical)	3.66	15.98	0	0	400	15.75
Degree, normal (generated)	3.72	0.93	4	0	6	−1.03
Degree, lockdown (empirical)	1.52	5.49	0	0	100	11.51
Degree, lockdown (generated)	1.56	1.17	1	0	6	0.52
Community and Social Service Occupations						
Share of the labor market (empirical)	0.01					
Share of the labor market (generated)	0.01	0	0.01	0.01	0.02	0.24
Degree, normal (empirical)	2.84	8.85	0	0	100	7.43
Degree, normal (generated)	2.91	0.66	3	0	5	−1.46
Degree, lockdown (empirical)	1.08	2.61	0	0	20	4.38
Degree, lockdown (generated)	1.11	0.95	1	0	4	0.51
Computer and Mathematical Occupations						
Share of the labor market (empirical)	0.02					
Share of the labor market (generated)	0.02	0	0.02	0.02	0.03	−0.24
Degree, normal (empirical)	2.85	7.77	0	0	75	5.34
Degree, normal (generated)	2.92	0.69	3	0	5	−1.26
Degree, lockdown (empirical)	1.21	3.89	0	0	54	7.2
Degree, lockdown (generated)	1.24	0.99	1	0	5	0.44

Table S3: Comparison of occupational groups in empirical versus generated networks, part 2.

	**Mean**	**SD**	**Median**	**Min**	**Max**	**Skew**
Construction and Extraction Occupations						
Share of the labor market (empirical)	0.03					
Share of the labor market (generated)	0.03	0	0.03	0.03	0.04	0.12
Degree, normal (empirical)	3.6	18.53	0	0	400	15.28
Degree, normal (generated)	3.67	0.91	4	0	6	−1.07
Degree, lockdown (empirical)	1.99	11.58	0	0	300	19.52
Degree, lockdown (generated)	2.05	1.25	2	0	6	0.18
Educational Instruction and Library Occupations						
Share of the labor market (empirical)	0.04					
Share of the labor market (generated)	0.04	0	0.04	0.04	0.05	−0.47
Degree, normal (empirical)	12.76	62.44	0	0	1100	12.45
Degree, normal (generated)	11.86	5.07	13	0	33	−0.31
Degree, lockdown (empirical)	2.61	18.87	0	0	500	20.5
Degree, lockdown (generated)	3.08	2.18	3	0	16	0.83
Farming, Fishing, and Forestry Occupations						
Share of the labor market (empirical)	0.01					
Share of the labor market (generated)	0	0	0	0	0.01	−0.36
Degree, normal (empirical)	2.15	4.98	0	0	36	3.75
Degree, normal (generated)	2.23	0.79	2	0	4	−0.45
Degree, lockdown (empirical)	1.76	4.98	0	0	50	5.72
Degree, lockdown (generated)	1.7	0.81	2	0	4	−0.07
Food Preparation and Serving Related Occupations						
Share of the labor market (empirical)	0.06					
Share of the labor market (generated)	0.06	0	0.06	0.06	0.07	−0.01
Degree, normal (empirical)	5.32	22.29	0	0	300	9.43
Degree, normal (generated)	5.32	1.43	6	0	9	−1.26
Degree, lockdown (empirical)	1.57	5.86	0	0	80	8.41
Degree, lockdown (generated)	1.62	1.32	1	0	8	0.78
Healthcare Practitioners and Technical Occupations						
Share of the labor market (empirical)	0.04					
Share of the labor market (generated)	0.04	0	0.04	0.04	0.05	−0.38
Degree, normal (empirical)	20.17	256.51	0	0	5000	19.07
Degree, normal (generated)	15.5	9.02	14	0	97	0.93
Degree, lockdown (empirical)	5.19	23.75	0	0	440	11.49
Degree, lockdown (generated)	5.34	3.49	5	0	40	1.05
Healthcare Support Occupations						
Share of the labor market (empirical)	0.03					
Share of the labor market (generated)	0.03	0	0.03	0.03	0.04	0.03
Degree, normal (empirical)	5.44	19.44	0	0	200	6.62
Degree, normal (generated)	5.46	1.43	6	0	9	−1.34
Degree, lockdown (empirical)	3.7	12.65	0	0	100	5.61
Degree, lockdown (generated)	3.81	1.57	4	0	9	−0.11

Table S4: Comparison of occupational groups in empirical versus generated networks, part 3.

	**Mean**	**SD**	**Median**	**Min**	**Max**	**Skew**
Installation, Maintenance, and Repair Occupations						
Share of the labor market (empirical)	0.03					
Share of the labor market (generated)	0.03	0	0.03	0.02	0.03	0.17
Degree, normal (empirical)	3.76	12.36	0	0	200	8.04
Degree, normal (generated)	3.82	0.95	4	0	6	−1.15
Degree, lockdown (empirical)	2.83	11.24	0	0	200	10.1
Degree, lockdown (generated)	2.91	1.18	3	0	6	−0.3
Legal Occupations						
Share of the labor market (empirical)	0.01					
Share of the labor market (generated)	0.01	0	0.01	0	0.01	0.2
Degree, normal (empirical)	8.28	22.06	0	0	110	3.38
Degree, normal (generated)	8.13	2.22	9	0	14	−1.61
Degree, lockdown (empirical)	0.92	2.98	0	0	28	6.73
Degree, lockdown (generated)	1.84	1.67	1	0	10	1.1
Life, Physical, and Social Science Occupations						
Share of the labor market (empirical)	0.01					
Share of the labor market (generated)	0.01	0	0.01	0	0.01	0.21
Degree, normal (empirical)	4.63	14.67	0	0	200	6.4
Degree, normal (generated)	4.67	1.15	5	0	7	−1.06
Degree, lockdown (empirical)	3.55	18.64	0	0	300	13.06
Degree, lockdown (generated)	3.65	1.34	4	0	7	−0.27
Management Occupations						
Share of the labor market (empirical)	0.05					
Share of the labor market (generated)	0.05	0	0.05	0.04	0.05	−0.37
Degree, normal (empirical)	5.84	23.54	0	0	500	10.79
Degree, normal (generated)	5.83	1.54	6	0	10	−1.4
Degree, lockdown (empirical)	1.32	4.79	0	0	100	9.7
Degree, lockdown (generated)	1.56	1.34	1	0	8	0.89
Office and Administrative Support Occupations						
Share of the labor market (empirical)	0.09					
Share of the labor market (generated)	0.1	0	0.09	0.09	0.1	−0.29
Degree, normal (empirical)	4.42	16.51	0	0	300	9.91
Degree, normal (generated)	4.46	1.15	5	0	7	−1.15
Degree, lockdown (empirical)	1.94	7.66	0	0	100	9.04
Degree, lockdown (generated)	2	1.37	2	0	7	0.52
Personal Care and Service Occupations						
Share of the labor market (empirical)	0.02					
Share of the labor market (generated)	0.02	0	0.02	0.02	0.03	−0.06
Degree, normal (empirical)	12.82	79.3	0	0	1000	10.31
Degree, normal (generated)	11.84	5.05	13	0	29	−0.28
Degree, lockdown (empirical)	4.58	41.84	0	0	891	17.9
Degree, lockdown (generated)	4.71	2.89	4	0	24	0.72

Table S5: Comparison of occupational groups in empirical versus generated networks, part 4.

	Mean	SD	Median	Min	Max	Skew
Production Occupations						
Share of the labor market (empirical)	0.04					
Share of the labor market (generated)	0.04	0	0.04	0.04	0.05	0.2
Degree, normal (empirical)	2.87	9.51	0	0	100	6.54
Degree, normal (generated)	2.91	0.67	3	0	5	−1.37
Degree, lockdown (empirical)	2.19	8.86	0	0	200	12.15
Degree, lockdown (generated)	2.25	0.93	2	0	5	−0.44
Protective Service Occupations						
Share of the labor market (empirical)	0.02					
Share of the labor market (generated)	0.02	0	0.02	0.01	0.02	0.3
Degree, normal (empirical)	1.12	3.3	0	0	20	3.83
Degree, normal (generated)	0.99	0.1	1	0	1	−10.33
Degree, lockdown (empirical)	1.05	2.37	0	0	10	2.66
Degree, lockdown (generated)	0.99	0.1	1	0	1	−10.33
Sales and Related Occupations						
Share of the labor market (empirical)	0.07					
Share of the labor market (generated)	0.07	0	0.07	0.06	0.08	−0.11
Degree, normal (empirical)	5.59	16.91	0	0	210	6.75
Degree, normal (generated)	5.58	1.45	6	0	9	−1.4
Degree, lockdown (empirical)	3.3	25.59	0	0	500	16.87
Degree, lockdown (generated)	3.4	1.71	3	0	9	0.07
Transportation and Material Moving Occupations						
Share of the labor market (empirical)	0.06					
Share of the labor market (generated)	0.06	0	0.06	0.06	0.06	−0.07
Degree, normal (empirical)	5.31	21.69	0	0	400	11.17
Degree, normal (generated)	5.3	1.43	6	0	9	−1.22
Degree, lockdown (empirical)	2.69	9.08	0	0	100	6.91
Degree, lockdown (generated)	2.77	1.65	3	0	9	0.36
Unemployed						
Share of the labor market (empirical)	0.05					
Share of the labor market (generated)	0.05	0	0.05	0.04	0.05	−0.09
Degree, normal (empirical)	2.34	12.45	NA	NA	NA	NA
Degree, normal (generated)	2.61	0.7	3	0	4	−1.35
Degree, lockdown (empirical)	0.96	5.08	NA	NA	NA	NA
Degree, lockdown (generated)	0.99	0.88	1	0	4	0.54
Retired						
Share of the labor market (empirical)	0.21					
Share of the labor market (generated)	0.21	0	0.21	0.2	0.22	0.11
Degree, normal (empirical)	2.13	12.56	NA	NA	NA	NA
Degree, normal (generated)	2.47	0.69	3	0	4	−0.94
Degree, lockdown (empirical)	0.87	5.12	NA	NA	NA	NA
Degree, lockdown (generated)	0.9	0.84	1	0	4	0.58

Table S6: Final sizes of the simulated epidemics by conditions and controls.

	**Baseline**			**Random**			**Targeted**		
	Mean	SD	Median	Mean	SD	Median	Mean	SD	Median
I. No lockdown									
Overall	90.97	19.07	95.48	60.78	29.98	69.16	38.58	37.24	38.87
Vaccine availability 5%				85.24	21.21	91.18	79.7	25.94	89.35
Vaccine availability 10%				79.7	22.38	86.46	65.72	30.39	80.01
Vaccine availability 20%				68.47	23.48	76.07	43.26	31.74	57.91
Vaccine availability 30%				55.7	24.95	64.63	23.66	27.3	0.48
Vaccine availability 40%				43.85	24.21	52.41	12.31	20.07	0.07
Vaccine availability 50%				31.74	22.6	38.75	6.84	14.09	0.04
Vaccine effectivity 60%				67.22	27.11	74.44	50.65	34.58	60.76
Vaccine effectivity 75%				60.8	29.52	68.5	37.18	37.01	30.03
Vaccine effectivity 90%				54.33	31.73	62.27	27.92	36.52	0.13
Low av. degree (4.46-4.74)	84.91	24.05	90.16	53	31.96	62.65	28.18	34.29	0.26
Medium av. degree (4.74-5.02)	92.25	17.18	96.53	61.87	29.56	69.72	37.39	37.16	36.11
High av. degree (5.02-5.3)	93.16	16.82	95.69	64.14	28.4	72.71	45.73	37.4	54.46
Low clustering (0.21-0.33)	95.11	13.04	97.32	66.2	27.4	74.34	47.23	37.16	56.29
Medium clustering (0.33-0.44)	91.98	18.68	95.46	62.32	29.43	71.94	41.19	37.42	45.34
High clustering (0.44-0.56)	83.59	23.97	90	50.74	31.81	60.24	22.28	31.48	0.14
Low av. path length (6.38-7.58)	94.74	14.76	97.3	66.27	27.45	74.66	46.01	37.36	55.19
Medium av. path length (7.58-8.77)	89.79	19.99	95.17	58.95	30.5	67.21	37.22	36.95	35.92
High av. path length (8.77-9.97)	83.81	24.07	90.76	50.73	31.94	60.23	21.08	30.93	0.12
Low homophily (0.06-0.11)	89.16	20.99	95.04	58.38	30.86	67.65	33.11	36.19	5.38
Medium homophily (0.11-0.16)	95.16	15.3	97.6	67.99	26.22	75.82	49.32	36.94	59.42
High homophily (0.16-0.2)	92.9	16.21	95.49	63.04	28.89	72.41	44.73	37.44	52.78
II. Lockdown									
Overall	26.64	26.59	39.66	10.66	17.64	0.04	2.53	8.86	0.02
Vaccine availability 5%				22.69	23.92	0.2	10.63	16.81	0.06
Vaccine availability 10%				19.13	21.4	0.11	3.77	9.48	0.04
Vaccine availability 20%				11.73	16.14	0.05	0.62	3	0.02
Vaccine availability 30%				6.5	11.29	0.04	0.09	0.54	0.02
Vaccine availability 40%				2.87	6.91	0.03	0.05	0.18	0.02
Vaccine availability 50%				1.06	3.71	0.02	0.03	0.09	0.01
Vaccine effectivity 60%				12.67	18.79	0.05	3.87	11.01	0.03
Vaccine effectivity 75%				10.43	17.43	0.04	2.25	8.27	0.02
Vaccine effectivity 90%				8.9	16.4	0.03	1.48	6.55	0.02
Low av. degree (2.06-2.1)	25.6	26.11	0.35	9.79	16.93	0.04	2.2	8.2	0.02
Medium av. degree (2.1-2.13)	27.59	26.93	41.42	11.29	18.06	0.04	2.73	9.17	0.02
High av. degree (2.13-2.16)	26.05	27.45	0.18	12.24	19.16	0.05	3.56	11.1	0.02
Low clustering (0.06-0.1)	35.33	28.58	56.81	14.86	20.76	0.05	4.63	12.42	0.02
Medium clustering (0.1-0.14)	25.43	25.73	0.28	10.07	16.76	0.04	1.95	7.2	0.02
High clustering (0.14-0.18)	17.6	21.5	0.09	6.3	12.85	0.04	0.79	3.83	0.02
Low av. path length (11.28-13.62)	34.05	28.55	54.69	14.48	20.46	0.05	4.42	12.07	0.02
Medium av. path length (13.62-15.97)	23.28	24.69	0.18	8.83	15.66	0.04	1.52	6.19	0.02
High av. path length (15.97-18.31)	15.28	20.32	0.08	5.48	11.89	0.04	0.59	3.04	0.02
Low homophily (0.04-0.06)	23.62	25.61	0.15	9.59	16.73	0.04	2.15	8.01	0.02
Medium homophily (0.06-0.09)	33.57	28.76	56.94	14.68	20.59	0.05	4.75	12.54	0.02
High homophily (0.09-0.11)	29.06	26.6	48.48	10.87	17.59	0.04	2.27	8.25	0.02

Table S7: Peak sizes of the simulated epidemics by conditions and controls.

	**Baseline**			**Random**			**Targeted**		
	Mean	SD	Median	Mean	SD	Median	Mean	SD	Median
I. No lockdown									
Overall	66.91	16.1	69.84	39.14	22.77	42.08	17.99	20.69	9.15
Vaccine availability 5%				60.76	17.04	64.18	46.95	18.16	51.8
Vaccine availability 10%				54.93	17.25	58.84	28.98	16.94	32.99
Vaccine availability 20%				43.7	16.82	47.65	15.22	13.86	14.76
Vaccine availability 30%				32.72	16.36	36.8	7.7	10.39	0.21
Vaccine availability 40%				23.6	14.68	26.68	3.86	6.98	0.05
Vaccine availability 50%				15.53	12.55	16.16	2.11	4.67	0.03
Vaccine effectivity 60%				43.86	21.03	46.61	24.6	20.92	22.09
Vaccine effectivity 75%				38.81	22.42	40.91	16.76	20.23	6.41
Vaccine effectivity 90%				34.13	23.51	34.87	12.03	18.47	0.09
Low av. degree (4.46-4.74)	56.22	17.59	58.47	30.03	21.03	31.95	11.63	16.71	0.17
Medium av. degree (4.74-5.02)	68.25	14.18	72.04	39.69	22.39	43.05	17.34	20.41	7.75
High av. degree (5.02-5.3)	71.71	14.1	71.18	43.91	22.33	47.26	22.38	21.8	18.52
Low clustering (0.21-0.33)	76.64	11.06	78.54	47.31	22.73	51.31	24.7	23.33	19.77
Medium clustering (0.33-0.44)	66.8	13.94	69.19	38.93	21.38	42.23	18.01	19.55	10.75
High clustering (0.44-0.56)	53.87	16.08	59.23	27.5	19.77	29	8.14	13.19	0.1
Low av. path length (6.38-7.58)	75.82	12.24	77.84	46.71	22.6	50.62	23.56	23	18.67
Medium av. path length (7.58-8.77)	63.19	14.78	67.53	35.62	21.28	38.45	15.81	18.57	7.57
High av. path length (8.77-9.97)	53.19	16.02	59.48	26.79	19.57	28.12	7.36	12.41	0.09
Low homophily (0.06-0.11)	63.25	17.04	65.12	36.15	22.48	38.97	15.03	19.23	3.24
Medium homophily (0.11-0.16)	79.42	12.74	81.43	50.66	22.51	55.48	26.29	23.8	21.78
High homophily (0.16-0.2)	70.12	13.15	70.36	41.68	21.97	44.89	20.92	21.13	16.09
II. Lockdown									
Overall	8.84	9.35	8.68	3.09	5.54	0.04	0.53	1.93	0.02
Vaccine availability 5%				7.06	7.93	0.13	2.15	3.77	0.05
Vaccine availability 10%				5.58	6.7	0.08	0.66	1.74	0.03
Vaccine availability 20%				2.99	4.45	0.04	0.13	0.46	0.02
Vaccine availability 30%				1.47	2.73	0.03	0.04	0.09	0.02
Vaccine availability 40%				0.59	1.45	0.03	0.03	0.05	0.02
Vaccine availability 50%				0.22	0.7	0.02	0.02	0.04	0.01
Vaccine effectivity 60%				3.65	5.93	0.04	0.83	2.5	0.02
Vaccine effectivity 75%				2.98	5.41	0.04	0.45	1.7	0.02
Vaccine effectivity 90%				2.52	5.01	0.03	0.28	1.23	0.02
Low av. degree (2.06-2.1)	8.17	8.82	0.22	2.73	5.12	0.04	0.43	1.66	0.02
Medium av. degree (2.1-2.13)	9.38	9.63	9.65	3.21	5.63	0.04	0.56	1.98	0.02
High av. degree (2.13-2.16)	9.4	10.69	0.15	3.76	6.6	0.04	0.84	2.79	0.02
Low clustering (0.06-0.1)	13.47	11.09	19.59	4.82	7.22	0.04	1.03	2.89	0.02
Medium clustering (0.1-0.14)	7.8	7.97	0.16	2.67	4.63	0.04	0.34	1.22	0.02
High clustering (0.14-0.18)	4.63	5.72	0.07	1.43	2.99	0.03	0.15	0.59	0.02
Low av. path length (11.28-13.62)	13	11.05	18.66	4.58	7	0.04	0.96	2.75	0.02
Medium av. path length (13.62-15.97)	6.78	7.25	0.11	2.26	4.14	0.04	0.26	0.99	0.02
High av. path length (15.97-18.31)	3.6	4.82	0.06	1.12	2.48	0.03	0.12	0.44	0.02
Low homophily (0.04-0.06)	7.64	8.82	0.09	2.72	5.12	0.04	0.47	1.78	0.02
Medium homophily (0.06-0.09)	13.4	11.69	22.14	4.87	7.41	0.04	1.06	2.97	0.02
High homophily (0.09-0.11)	9.06	8.5	13.21	2.95	5.04	0.04	0.39	1.43	0.02

Table S8: Mean peak size of baseline condition and difference by test condition in percent points.

	Baseline	Random	Targeted	
		to Baseline	to Baseline	to Random
I. No lockdown				
Overall	66.91	−27.77	−48.92	−21.15
Vaccine availability 5%		−6.16	−19.97	−13.81
Vaccine availability 10%		−11.99	−37.94	−25.95
Vaccine availability 20%		−23.21	−51.69	−28.48
Vaccine availability 30%		−34.19	−59.21	−25.02
Vaccine availability 40%		−43.32	−63.05	−19.73
Vaccine availability 50%		−51.38	−64.81	−13.43
Vaccine effectivity 60%		−23.05	−42.31	−19.26
Vaccine effectivity 75%		−28.10	−50.15	−22.05
Vaccine effectivity 90%		−32.78	−54.89	−22.11
Low clustering	76.64	−29.33	−51.94	−22.61
Medium clustering	66.80	−27.87	−48.79	−20.92
High clustering	53.87	−26.37	−45.72	−19.36
Low homophily	63.25	−27.10	−48.22	−21.12
Medium homophily	79.42	−28.76	−53.13	−24.37
High homophily	70.12	−28.44	−49.20	−20.76
II. Lockdown				
Overall	8.84	−5.75	−8.31	−2.56
Vaccine availability 5%		−1.78	−6.70	−4.92
Vaccine availability 10%		−3.26	−8.18	−4.92
Vaccine availability 20%		−5.85	−8.72	−2.87
Vaccine availability 30%		−7.38	−8.81	−1.43
Vaccine availability 40%		−8.25	−8.81	−0.56
Vaccine availability 50%		−8.62	−8.82	−0.20
Vaccine effectivity 60%		−5.20	−8.02	−2.82
Vaccine effectivity 75%		−5.87	−8.39	−2.53
Vaccine effectivity 90%		−6.33	−8.57	−2.24
Low clustering	13.47	−8.65	−12.44	−3.79
Medium clustering	7.80	−5.13	−7.46	−2.32
High clustering	4.63	−3.20	−4.48	−1.29
Low homophily	7.64	−4.92	−7.17	−2.25
Medium homophily	13.40	−8.53	−12.34	−3.81
High homophily	9.06	−6.11	−8.67	−2.56

Table S9: Duration of the simulated epidemics by conditions and controls.

	Baseline			Random			Targeted		
	Mean	SD	Median	Mean	SD	Median	Mean	SD	Median
I. No lockdown									
Overall	61.47	13.44	62	68.27	27.21	72	76.81	60.26	76
Vaccine availability 5%				63.18	15.68	64	75.07	24.21	77
Vaccine availability 10%				64.91	17.49	67	95.64	46.55	97
Vaccine availability 20%				68.58	21.53	72	101.85	68.39	106
Vaccine availability 30%				70.19	27.08	76	83.43	73.09	59
Vaccine availability 40%				71.81	32.63	80	59.01	61.46	25
Vaccine availability 50%				70.93	39.61	82	45.85	54.19	19
Vaccine effectivity 60%				68.02	23.47	71	82.98	46.59	89
Vaccine effectivity 75%				68.61	26.87	72	79.28	61.14	77
Vaccine effectivity 90%				68.16	30.8	72	68.16	69.76	33
Low av. degree (4.46-4.74)	64.62	16.03	67	70.03	32.45	76	69.77	62.9	41
Medium av. degree (4.74-5.02)	61.74	12.72	62	69.33	26.94	72	76.44	61.19	75
High av. degree (5.02-5.3)	59.4	12.1	60	66.19	23.86	69	81.2	57.32	81
Low clustering (0.21-0.33)	55.57	8.6	56	62.58	20.99	65	76.95	53.31	77
Medium clustering (0.33-0.44)	62.89	12.69	64	71.04	26.63	75	83.57	62.62	84
High clustering (0.44-0.56)	67.07	16.69	69	71.22	33.72	78	64.94	63.14	30
Low av. path length (6.38-7.58)	55.96	9.3	56	63.86	21.58	66	77.59	54.81	77
Medium av. path length (7.58-8.77)	64.03	13.71	65	70.74	28.49	75	79.65	62.92	80
High av. path length (8.77-9.97)	69.05	16.34	71.5	72.85	34.86	80	64.53	64.68	28
Low homophily (0.06-0.11)	62.94	14.47	64	69.92	29.35	74	73.56	62.2	67
Medium homophily (0.11-0.16)	53.36	9.07	53	59.95	18.47	62	75.7	49.6	76
High homophily (0.16-0.2)	60.75	11.86	61	67.3	24.82	70	81.79	58.73	82
II. Lockdown									
Overall	72	57.25	91.5	57.43	63.59	19	33.73	54.58	14
Vaccine availability 5%				72.29	61.08	39	73.85	85.93	22
Vaccine availability 10%				71.82	63.82	30	51.92	73.55	19
Vaccine availability 20%				66.53	69.17	22	28.01	40.84	15
Vaccine availability 30%				57.56	68.47	19	18.08	16.88	12
Vaccine availability 40%				44.62	60.36	16	16.18	12.34	11
Vaccine availability 50%				31.79	44.85	14	14.36	8.64	10
Vaccine effectivity 60%				62.7	65.31	21	40.76	60.53	16
Vaccine effectivity 75%				57.22	63.3	19	32.35	52.51	14
Vaccine effectivity 90%				52.38	61.69	18	28.09	49.33	12
Low av. degree (2.06-2.1)	72.86	58.6	55.5	55.55	62.89	19	32.73	54.07	14
Medium av. degree (2.1-2.13)	71.79	56.27	94	58.98	64.22	20	34.47	55.16	14
High av. degree (2.13-2.16)	65.7	55.01	36.5	58.46	62.83	20	35.3	52.78	14
Low clustering (0.06-0.1)	74.33	50.61	99	61.86	62.38	21	40.62	62.4	14
Medium clustering (0.1-0.14)	73.1	58.59	46.5	58.13	64.6	19	33.07	54.87	14
High clustering (0.14-0.18)	67.43	62.65	26	50.85	63.03	18	26.13	40.87	13
Low av. path length (11.28-13.62)	72.41	50.27	99	61.56	62.53	21	39.7	60.86	14
Medium av. path length (13.62-15.97)	72.7	60.21	38	55.81	64.05	19	30.94	51.77	14
High av. path length (15.97-18.31)	66.05	66.29	22	49.66	64.09	18	25.11	37.66	13
Low homophily (0.04-0.06)	68.68	58.08	34	55.88	64	19	31.4	49.59	13
Medium homophily (0.06-0.09)	69.83	48.59	96	60.11	60.03	21	40.57	61.97	14
High homophily (0.09-0.11)	78.86	58.67	109	59.06	64.25	20	34.98	59.23	14

Table S10: Mean duration of baseline condition and difference by test condition in percent points.

	Baseline	Random to Baseline	Targeted to Baseline	Targeted to Random
I. No lockdown				
Overall	61.47	6.80	15.34	8.54
Vaccine availability 5%		1.71	13.60	11.89
Vaccine availability 10%		3.44	34.17	30.73
Vaccine availability 20%		7.11	40.38	33.27
Vaccine availability 30%		8.72	21.96	13.24
Vaccine availability 40%		10.34	−2.46	−12.80
Vaccine availability 50%		9.46	−15.62	−25.08
Vaccine effectivity 60%		6.55	21.51	14.96
Vaccine effectivity 75%		7.14	17.81	10.67
Vaccine effectivity 90%		6.69	6.69	0.00
Low clustering	55.57	7.01	21.38	14.36
Medium clustering	62.89	8.15	20.68	12.53
High clustering	67.07	4.16	−2.13	−6.29
Low homophily	62.94	6.99	10.62	3.63
Medium homophily	53.36	6.59	22.34	15.75
High homophily	60.75	6.55	21.04	14.49
II. Lockdown				
Overall	72.00	−14.56	−38.26	−23.70
Vaccine availability 5%		0.29	1.85	1.57
Vaccine availability 10%		−0.18	−20.07	−19.89
Vaccine availability 20%		−5.47	−43.99	−38.52
Vaccine availability 30%		−14.43	−53.92	−39.48
Vaccine availability 40%		−27.38	−55.81	−28.44
Vaccine availability 50%		−40.21	−57.64	−17.43
Vaccine effectivity 60%		−9.30	−31.24	−21.94
Vaccine effectivity 75%		−14.78	−39.65	−24.87
Vaccine effectivity 90%		−19.61	−43.90	−24.29
Low clustering	74.33	−12.47	−33.72	−21.24
Medium clustering	73.10	−14.97	−40.03	−25.06
High clustering	67.43	−16.57	−41.30	−24.72
Low homophily	68.68	−12.79	−37.28	−24.48
Medium homophily	69.83	−9.72	−29.26	−19.54
High homophily	78.86	−19.80	−43.88	−24.08

Supplementary Methods

Note that the following elaborations are partly taken from an unpublished manuscript [1] that presents the model used for network generation and simulation of epidemics. It is a specific model case of the *Networking during Infectious Disease Model* (*NIDM*) [2], designed to simulate epidemics in small-world networks with control over degree per node and network clustering. The information provided is complete in terms of components used for the current study.

Network Generation

Network generation is based on utility maximization for network positions. That is, utility for an agent i is a trade-off between benefits (B_i) and maintenance costs (C_i) a social tie creates:

$$U_i(\boldsymbol{G}) = B_i(\boldsymbol{G}) - C_i(\boldsymbol{G}). \tag{1}$$

We consider networks to be undirected, unweighted, and non-reflexive graphs:

$$\boldsymbol{G} = \begin{bmatrix} g_{11} & g_{12} & \cdots & g_{1N} \\ g_{21} & g_{22} & \cdots & g_{2N} \\ \vdots & \vdots & \ddots & \vdots \\ g_{N1} & g_{N2} & \cdots & g_{NN} \end{bmatrix}, \tag{2}$$

with $g_{ij} \in \{0,1\}$, $g_{ii} = 0$, and $g_{ij} = g_{ji} = 1$ if a tie exists. Social benefit is defined as the sum of weighted benefits for direct ties and weighted benefits for the proportion of closed triads of agent i:

$$B_i(\boldsymbol{G}) = b_1 \cdot t_i + b_2 \cdot \left(1 - 2 \cdot \frac{|x_i - \alpha|}{\max(\alpha, 1-\alpha)}\right), \tag{3}$$

with x_i denoting the actual proportion of closed triads i belongs to in i's ego-network, α the preferred proportion of closed triads of agent i, and t_i the number of ties agent i possesses:

$$t_i = \sum_j g_{ij}. \tag{4}$$

Social maintenance costs are assumed to be quadratic in the number of ties t_i to model increasing marginal costs of additional ties:

$$C_i(\boldsymbol{G}) = c_1 \cdot t_i + c_2 \cdot t_i^2. \tag{5}$$

Network generation for reported contact numbers prior to lockdown was realized using the NIDM network formation procedure. That is, time is modeled as discrete time steps. Within each time step, we simulate the formation and dissolution of ties:

- Create an empty set of unprocessed agents A[1] and repeat until all agents have been processed:
  - Randomly select an unprocessed agent i, either:
    - from A if $A \neq \{\}$ and not all agents from A have been processed yet, or
    - from the entire population N if $A = \{\}$.
  - Repeat until agent i has evaluated $\phi \cdot (N-1)$ ties other agents j:
    - Create a new set J of agents to be evaluated through one of the three following options:
      1. With probability ψ: J becomes all agents at distance 1 that have not yet been evaluated by i in the current time step.
      2. With probability ξ: J becomes all agents at distance 2 that have not yet been evaluated by i in the current time step.
      3. With probability $1 - \psi - \xi$: J becomes all agents from the entire population that have not yet been evaluated by i in the current time step.

[1] The use of A (in combination with parameters ψ and ξ that are introduced later) and thus the consecutive processing of an agent's neighbors allows producing high levels of clustering and homophilous mixing in networks too large for a purely randomized approach.

        * Select a single agent j from J either:
            · Belonging to the same occupational group (with probability ω), or
            · a randomly selected agent (with probability $1 - \omega$).
        * If j is directly tied to i:
            · Dissolve tie ij, if i's utility excluding ij exceeds the utility including ij.
        * else:
            · Form tie ij, if both i's and j's utilities including ij exceed the utilities excluding ij.
        * Add j to A if j is directly tied to i.

Generation of lockdown networks was realized using a network pruning algorithm:

- For all networks N:
    - Repeat until N is "in lockdown" (av. degree of all occupational groups $\leq$ reported av. lockdown degree + 3%):
        * For all edges e connecting nodes n_1 and n_2 in N (in random order):
            · If n_1 and n_2 are not yet "in lockdown" (degree > reported av. lockdown degree of occupational group + 3%):
                · Remove e with a probability depending on the reported percentage decrease in degree between prior and during lockdown (t^-): $\frac{t^-_{n_1} + t^-_{n_2}}{2}$

Disease Spread

Disease states (susceptible, infected, recovered, or vaccinated) are defined for each node in the network:

$$\boldsymbol{d} \in \{S, I, R, V\}^N. \tag{6}$$

The probability for a node i to get infected per time step

$$\pi_i(\boldsymbol{G}, \boldsymbol{d}) = 1 - (1 - \gamma)^{t_{i_I}}, \tag{7}$$

depends on the probability to get infected per single contact (γ) and the number of infected neighbors:

$$t_{i_I} = \sum_{j,\ d_j=I} g_{ij}. \tag{8}$$

As before, time is modeled as discrete time steps. Within each time step, we simulate disease transmission events:

- Repeat until all nodes have been processed:
  - Randomly select an unprocessed node i:
  - If i is infected, compute whether node recovers: passed time steps since infection $\geq \tau$.
  - If i is susceptible, compute whether i gets infected from infected neighbors (see Equation 7).

Parameters and Submodels

Table S11 presents all parameters including the range of possible settings and the initial settings used for network generation and the simulation of epidemics. We systematically varied the settings for α (to control clustering) and ω (to control occupational group homophily) and selected for each combination the 10 best fits, resulting in 90 normal (prior to lockdown) networks. Afterwards, we used the pruning algorithm described earlier to generate 90 lockdown networks.

Based on these 180 networks, we simulated 20 epidemics for each of the conditions (Baseline, Random, Targeted). For the two vaccination campaign conditions, we systematically varied the vaccine parameters (availability, effectivity) to end up with $1 \cdot 20 \cdot 180 + 2 \cdot 20 \cdot 180 \cdot 6 \cdot 3 = 133,200$ simulated epidemics.

Network size was selected to ensure large enough networks to make meaningful inferences, while limiting computational demands to allow a large number of network variations and simulated epidemics. Other fixed parameters were selected because of one of two reasons. First, they are backed by empirical data. Recent US labor market numbers were taken to set occupational group size [3]. Clustering in contact networks was found to be around 0.46 with clustering getting lower for older persons [4]. We, therefore, varied α

between 0.3, 0.4, and 0.5. Average degree per contact in our model can be realized in the model by keeping the b_1 and c_1 constant, and setting c_2 to define the optimum number of ties: $\frac{b_1 - c_1}{2 \cdot t_{\text{target}}}$. This is done for each node individually, dependent on reported contact numbers per occupational group. Other fixed parameters were selected because pilot runs showed that they produce informative and interesting variations of epidemics regarding final size, epidemic peak size, and epidemic peak time.

Table S11: Parameters, ranges, and initial settings.

Parameter	Range	Initial setting
I. Network Generation		
I.I. Utility		
Benefit per social tie	$b_1 \in \mathbb{R}_0^+$	$b_1 = 1.0$
Benefit for triadic closure	$b_2 \in \mathbb{R}_0^+$	$b_2 = 0.5$
Preferred proportion of closed triads	$0 \leq \alpha \leq 1$	$\alpha \in \{0.3, 0.4, 0.5\}$
Simple cost per tie	$c_1 \in \mathbb{R}_0^+$	$c_1 = 0.2$
Marginal cost per tie	$c_2 \in \mathbb{R}_0^+$	$c_2 = [0.02, 0.36]$*
I.II. Network		
Number of agents	$N \in \mathbb{Z}_0^+$	$N = 10000$
I.III. Simulation		
Proportion of nodes offered to evaluate per time step	$0 \leq \phi \leq 1$	$\phi = 0.001$
Proportion of ϕ as distance 1 ties	$0 \leq \psi \leq 1$	$\psi = 0.3$
Proportion of ϕ as distance 2 ties	$0 \leq \xi \leq 1$	$\xi = 0.5$
Likelihood of offered alter from the same occ. group	$0 \leq \omega \leq 1$	$\omega \in \{0.0, 0.4, 0.8\}$
**II. Simulation of Epidemics**		
II.I. Disease		
Probability of disease transmission per time step	$0 \leq \gamma \leq 1$	$\gamma = 0.15$
Recovery time in time steps	$\tau \in \mathbb{Z}^+$	$\tau = 10$
II.II. Vaccination		
Vaccine availibility	$0 \leq \theta \leq 1$	$\theta \in \{0.05, 0.1, 0.2, 0.3, 0.4, 0.5\}$
Vaccine effectivity	$0 \leq \eta \leq 1$	$\eta \in \{0.6, 0.75, 0.9\}$

*Settings for c_2 depended on occupational group membership and reported contact numbers. Healthcare Practioners and Technical Occupations, for example, had the highest reported contact numbers (20.17), thus receiving the lowest marginal costs per tie: $c_2 = \frac{1.0-0.2}{2 \cdot 20.17} = 0.02$. Protective Service Occupations, on the other hand, had the lowest reported contact numbers (1.12), thus receiving the highest marginal costs per tie: $c_2 = \frac{1.0-0.2}{2 \cdot 1.12} = 0.36$.

References

[1] Hendrik Nunner, Vincent Buskens, Alexandra Teslya, and Mirjam Kretzschmar. The effects of health behavior homophily on epidemics in adaptive and static small-world networks. 2021.

[2] Hendrik Nunner, Vincent Buskens, and Mirjam Kretzschmar. A model for the co-evolution of dynamic social networks and infectious disease dynamics. *Computational Social Networks*, 8(1):19, 2021. doi: 10.1186/S40649-021-00098-9.

[3] U.S. Bureau of Labor Statistics. Employment by major occupational group, 2019 and projected 2029, 2019. URL `https://www.bls.gov/emp/tables/emp-by-major-occupational-group.htm`.

[4] Leon Danon, Jonathan M. Read, Thomas A. House, Matthew C. Vernon, and Matt J. Keeling. Social encounter networks: characterizing Great Britain. *Proceedings of the Royal Society B: Biological Sciences*, 280(1765):20131037, 2013. doi: 10.1098/rspb.2013.1037.
